# Integrated Physio-Biochemistry and Transcriptome Analysis Reveals the Mechanism of 24-Epibrassinolide in Alleviating Cadmium Stress in Watermelon (*Citrullus lanatus* L.)

**DOI:** 10.3390/biology15080638

**Published:** 2026-04-18

**Authors:** Jingqiu Xu, Yuanyuan Chen, Mengmeng Liu, Haidong Ding

**Affiliations:** College of Bioscience and Biotechnology, Yangzhou University, Yangzhou 225009, China

**Keywords:** antioxidant defense, cadmium, 24-epibrassinolide, phenylpropanoid pathway, transcriptome, *Citrullus lanatus*

## Abstract

Cadmium (Cd) contamination is widely recognized as a major risk factor affecting the security and quality of crop production. Watermelon (*Citrullus lanatus*) is a globally cultivated fruit that is susceptible to Cd stress. 24-Epibrassinolide (EBR), an active brassinosteroid, is essential for plant growth and abiotic stress responses. However, its protective role in watermelon under Cd stress remains unclear. This study elucidates the physiological and molecular processes underlying EBR-mediated alleviation of Cd toxicity in watermelon seedlings. The results showed that exogenous EBR application effectively mitigated Cd-induced growth inhibition through decreased Cd deposition, reduced the accumulation of reactive oxygen species (ROS), lowered membrane lipid peroxidation, and increased antioxidant capacity in watermelon leaves under Cd treatment. Transcriptome (RNA-Seq) analysis revealed that EBR triggered substantial reprogramming of gene expression patterns, identifying 530 differentially expressed genes (DEGs) in Cd + EBR co-treatment compared with Cd treatment alone, including 204 down-regulated genes and 326 up-regulated genes. These DEGs are vital for controlling several physiological processes, including phenylpropane metabolism, phenylpropanoid biosynthesis, endoplasmic reticulum’s protein production, cell wall organization, and others. Further physiological assays confirmed that EBR increased the activities of PAL and 4CL, the core enzymes driving phenylpropanoid biosynthesis, leading to a significant accumulation of total phenols and flavonoids. Together, the above results give concrete proof of the powerful functions of 24-EBR, acting as an enhancer of plant performance under Cd stress by enhancing the antioxidant system and by activating the phenylpropanoid pathway and its derived metabolic networks.

## Simple Summary

Cadmium pollution harms crop safety and quality. This study shows that applying a natural plant hormone, 24-epibrassinolide, helps watermelon seedlings to survive cadmium stress. The hormone works by reducing cadmium accumulation, easing oxidative damage, and boosting the plants’ antioxidant defenses. Gene analysis showed that the hormone turned on protective genes, especially those controlling the production of natural antioxidants (phenols and flavonoids). This research reveals how the hormone strengthens watermelon’s defense against cadmium and provides a basis for breeding heavy-metal-tolerant watermelon cultivars.

## 1. Introduction

With rapid urbanization and industrial development, the widespread use of pesticides, fertilizers, and sewage irrigation, collectively known as the “three wastes” of industry, has led to increasing soil contamination by heavy metals [1]. Cadmium (Cd) has no essential biological function but exhibits high toxicity, mobility, and bioaccumulation potential, making it a major environmental pollutant threatening agricultural ecosystems [2,3]. It suppresses seed germination, reduces plant growth, and disrupts photosynthesis by degrading chlorophyll and damaging the photosynthetic apparatus. It also combines with sulfhydryl and carboxyl groups, thereby affecting enzyme activity [4,5]. Moreover, Cd can be transferred along the food chain through contaminated crops, posing serious risks to human health, such as kidney damage, bone disease, and carcinogenesis.

Using exogenous substances to treat plants is currently one of the simplest and most feasible methods for improving plant stress resistance. 24-Epibrassinolide (EBR), a highly active brassinosteroid, regulates gene expression and physiological metabolism during seed germination, photosynthesis, reproductive growth, and stress response [6,7]. The application of EBR as a plant regulator has greatly reduced the toxicological effects of heavy metals [3,6,8,9]. For instance, under Cr and Pb stress, EBR acts as a growth promoter in tomato plants by modulating physiological, metabolic, and defense systems [7,8]. Regarding Cd, BR application also alleviates Cd toxicity in various plants such as *Solanum nigrum* [2] and *Kentucky bluegrass* [10]. Recently, it was reported that EBR treatment enhances Cd tolerance in adzuki bean seedlings by modulating antioxidant defense, osmotic balance, Cd detoxification, DNA damage repair, and cell cycle progression [11]. However, the investigation of the molecular mechanisms through which EBR alleviates heavy metal stress, including Cd, is still in its infancy.

Watermelon (*Citrulus lanatus* L.) is an important horticultural crop which belongs to the Cucurbitaceae family with wide distribution throughout the world. Watermelons cultivated in soils contaminated with Cd typically accumulate much higher levels of Cd in their edible flesh compared to those grown in non-contaminated soils [12], thereby presenting a potential health hazard to consumers. The genome of watermelon has been sequenced [13,14], which is a valuable resource for the study of plant stress tolerance at the molecular level. Just two years ago, it was reported that Cd stress inhibited watermelon seedling growth, reduced leaf area, and induced ROS accumulation. The same study also showed that Cd stress triggered activation of the antioxidant system, glutathione metabolism and the mitogen-activated protein kinase signaling pathway in watermelon as adaptive defense mechanisms [15]. However, no studies to date have investigated EBR-mediated alleviation of Cd toxicity in watermelon, particularly at the molecular level.

This study combined physiological and transcriptomic approaches to explore how EBR alleviates Cd stress in watermelon seedlings. First, the optimal EBR concentration for alleviating Cd toxicity in watermelon was determined. Next, the role of 0.05 μM EBR in mitigating oxidative stress under Cd stress was further examined. Then, transcriptome sequencing of watermelon leaves was performed, followed by analysis of differentially expressed genes (DEGs). Finally, key tolerance-related genes were investigated to contribute a theoretical framework for elucidating plant defense mechanisms against Cd toxicity and to support the practical application of EBR.

## 2. Materials and Methods

### 2.1. Plant Materials, Stress Treatments and Experimental Design

The experimental work was executed in a controlled melon and vegetable greenhouse under controlled environmental conditions: day/night temperature regime—25/20 ± 2 °C; photoperiod—14/10 h; and relative humidity—70%. Uniformly plump seeds of a similar size were selected, soaked to facilitate germination, and sown in 10 × 10 cm plastic pots containing vermiculite. Seedlings received irrigation with quarter-strength Hoagland nutrient solution at two-day intervals.

At the seedling stage characterized by two fully expanded leaves and a central heart leaf, uniformly grown seedlings were selected for two independent experiments. In the first experiment, to screen for the optimal EBR concentration, seven treatments were established: control (no Cd, no EBR); T0.00 (0.2 mM Cd + 0 μM EBR); T0.025 (0.2 mM Cd + 0.025 μM EBR); T0.05 (0.2 mM Cd + 0.05 μM EBR); T0.10 (0.2 mM Cd + 0.10 μM EBR); T0.20 (0.2 mM Cd + 0.20 μM EBR); and T0.40 (0.2 mM Cd + 0.40 μM EBR). For the control group, the seedlings were neither sprayed with EBR nor watered with Cd. Their leaves were directly sprayed with distilled water until they were thoroughly wetted and droplets formed on the surface, and they were irrigated directly with nutrient solution. For the treatment groups (T0.00–T0.40), seedlings were sprayed with different concentrations of EBR one day prior to treatment, and then watered every three days with nutrient solution containing 0.2 mM Cd. After seven days of Cd/EBR application, all seedlings were harvested, and their fresh weight was recorded. Each treatment was randomly arranged with three replicates (pots). Cd was supplied via CdCl_2_·2.5H_2_O supplementation in the nutrient solution.

In a subsequent experiment, to investigate the physiological and transcriptomic mechanisms, four treatments were applied: control (no Cd, no EBR); EBR (0.05 µM EBR alone); Cd (0.2 mM Cd alone); and Cd + EBR (0.2 mM Cd + 0.05 µM EBR). At 7 days post-treatment, watermelon leaf samples were collected, snap-frozen in liquid nitrogen, and stored at −80 °C for downstream analysis.

### 2.2. Growth and Cd Accumulation

The seedlings were rinsed sequentially with tap water (3×) followed by distilled water (3×), then blotted dry with absorbent paper. The aboveground and belowground tissues of the sampled plant materials were separated. The fresh weight (FW) was determined first, then placed in an oven. The seedlings were fixed at 105 °C for 30 min, then dried at 80 °C and weighed; this procedure was repeated three times. Dried plant samples were digested in a 4:1 (*v*/*v*) mixture of nitric acid and perchloric acid for 12 h, until the solution became transparent. After filtration, the volume was adjusted to 50 mL, and the Cd levels in the aboveground portion were quantified by an ICP spectrometer (iCAP 6300) (Thermo Scientific, Waltham, MA, USA).

### 2.3. Chlorophyll Content

A 95% ethanol extraction was used to quantify the contents of chlorophyll a and b in seedling leaves and the total chlorophyll content was calculated as chlorophyll a plus chlorophyll b. The absorbance at wavelengths of 470, 649, and 665 nm was recorded and the content was measured according to the formulae [16]: Chlorophyll a = 13.95 × OD665 − 6.88 × OD649 and Chlorophyll b = 24.96 × OD649 − 7.32 × OD665.

### 2.4. H_2_O_2_ and MDA Content

The H_2_O_2_ and MDA contents were determined [17]. Briefly, to prepare plant extracts for analysis, fresh leaf samples (0.1 g) were homogenized in 5 mL of 5% (*w*/*v*) trichloroacetic acid (TCA). Samples were centrifuged at 12,000× *g* for 20 min at 4 °C, and the supernatant was collected. For H_2_O_2_ determination, the assay mixture (consisting of 0.1 mL supernatant, 0.5 mL of 1.0 M KI and 0.5 mL of 50 mM potassium phosphate buffer, pH 7.0) was prepared. The absorbance was read at 390 nm. The content of H_2_O_2_ was calculated from a standard curve. A thiobarbituric acid (TBA) assay was employed to identify the amount of malondialdehyde (MDA). Then, 1 mL of the supernatant was taken and mixed with 1 mL of 0.6% TBA solution. After thorough mixing, the mixture was heated in a boiling water bath for 20 min, then rapidly cooled to room temperature (an ice-water bath was used to terminate the reaction). Following cooling, the mixture was centrifuged at 12,000× *g* and 4 °C for 10 min. The resulting supernatant was collected, and its absorbance was measured at 532 nm, 600 nm, and 450 nm, using a spectrophotometer.

### 2.5. Activities of Antioxidant Enzymes

Fresh leaf samples (0.5 g) were grounded with 1 mM EDTA and 1% PVP in a phosphoric acid buffer solution (pH 7.8) under pre-cooling conditions and centrifuged for 30 min (4 °C, 12,000× *g*), and the supernatant was aliquoted and stored for subsequent analysis. According to the procedure [17], the capacity of superoxide dismutase (SOD) to suppress nitroblue tetrazolium (NBT) photochemical reduction was assessed. The 50 μL supernatant was mixed with a reaction buffer (50 mM phosphate buffer pH 7.0, 13 mM methionine, 75 mM NBT, 100 mM EDTA, and 2 mM riboflavin). The mixture was illuminated for 10 min, with the control kept in the dark. SOD activity (per unit) was quantified as the enzyme concentration required to inhibit NBT reduction by 50%, with absorbance measured at 560 nm. Catalase (CAT) activity was assayed by monitoring the decrease in absorbance at 240 nm. The reaction mixture contained 1 mL of 0.1 M PBS (pH 7.5), 0.2 mL of 0.1 M H_2_O_2_, and 50 μL of the supernatant. Enzyme activity was quantified based on the consumption of H_2_O_2_, using its molar extinction coefficient (ε = 45.2 mM^−1^ cm^−1^). Ascorbate peroxidase (APX) activity was assayed by monitoring the decrease in absorbance at 290 nm. The reaction mixture was prepared containing 1 mL of 0.1 M PBS (pH 7.5), 0.1 mL of 0.01 M H_2_O_2_, 0.1 mL of 5 mM ascorbate, and 50 μL of the supernatant. The decrease in absorbance of ascorbate at 290 nm was recorded, and the enzyme activity was quantified using the molar extinction coefficient of 2.8 mM^−1^ cm^−1^.

### 2.6. RNA-Seq

For RNA-seq analysis, two independent cDNA libraries were constructed from seedlings subjected to Cd alone (0.2 mM Cd) or combined Cd + EBR treatment (0.2 mM Cd + 0.05 µM EBR). Each treatment group comprised three biological replicates, yielding a total of six samples. The total RNA was extracted from plant tissues using a polysaccharide-polyphenol RNA extraction kit. Following the qualification of the samples’ purity and integrity, cDNA libraries were created. mRNA was enriched using Oligo(dT)-conjugated magnetic beads and randomly fragmented by the addition of Fragmentation Buffer. Using the fragmented mRNA as a template, first-strand cDNA was synthesized with random hexamers, followed by second-strand cDNA synthesis. The resulting double-stranded cDNA was purified with AMPure XP beads, then subjected to end repair, A-tailing, adapter ligation, and size selection using AMPure XP beads. Finally, the cDNA library was obtained by PCR enrichment. After the library was constructed and qualified, Illumina HiSeq TM2000 (San Diego, CA, USA) was used to perform transcriptome sequencing analysis on the RNA samples. The raw data were cleaned by fastp (v0.23.4) to remove low-quality information such as joints and unclear base information from the original data. The watermelon genome (http://www.watermelondb.cn) was used as a reference for the comparative analysis of the reference transcriptome. FPKM (fragments per kilobase of transcript per million fragments mapped) was used as the indicator to measure the transcript or gene expression levels. The calculation formula for FPKM is as follows:

$$FPKM=\frac{cDNA\;Framents}{Mapped\;Reads\;(Millions)\times Transcript\;Length\;(kb)}$$

DESeq2 software was used to analyze the DEGs (Cd + EBR vs. Cd). |log2 (FoldChange)| ≥ 1&padj ≤ 0.05 was used as the standard to screen the differential genes (DEGs). GO and KEGG enrichment analyses were conducted for DEGs using Cluster Profiler software.

### 2.7. qRT-PCR Verification

Real-time fluorescent quantitative PCR (qRT-PCR) validation was performed on ten randomly selected DEGs. The total RNA was extracted from watermelon leaves using a commercial kit, and reverse transcription was performed to synthesize cDNA. qRT-PCR was carried out using ChamQ™ SYBR^®^ qPCR Master Mix on a qRT-PCR instrument (BIO-RAD Applied Biosystems, Carlsbad, CA, USA), with watermelon *actin* as the housekeeping gene. The thermal cycling protocol was as follows: 95 °C; 15 min; and 40 PCR cycles (95 °C, 10 s; 58 °C, 30 s; 72 °C, 30 s). Fluorescence quantitative data were calculated by 2^−ΔΔCt^ relative determination. The primer sequences are seen in Table S1.

### 2.8. Determination of Total Flavonoids and Total Phenols

The total flavonoid content was determined with slight modifications [18]. A stock standard solution was prepared by dissolving a dried rutin standard in 60% ethanol. Rutin standard solutions of varying concentrations were prepared using the double-dilution method. Then, 2 mL of each standard solution was taken; 0.5 mL of 5% sodium nitrite, 0.5 mL of 10% aluminum nitrate, and 4.0 mL of 4% sodium hydroxide were added sequentially, and the volume was adjusted using 60% ethanol. Absorbance was measured at 510 nm. Then, 1 g of fresh leaves was combined with 6 mL of 60% ethanol, maintained in a 70 °C water bath for a duration of 60 min, and then filtered. The following steps were the same as the standard solution. The total flavonoid content was calculated based on the standard curve.

Fresh leaves (0.25 g) were accurately weighed, ground into a homogenate with 1.5 mL of 95% ethanol, mixed with an additional 2.5 mL of 95% ethanol, filtered, and diluted to 25 mL with 95% ethanol. Then, 1 mL of the prepared test solution was mixed with 1 mL of Folin–Ciocalteu reagent and 2 mL of 10% sodium carbonate solution, and left to stand for 1 h. The absorbance was measured at 760 nm and 1 mL of 95% was used as the blank ethanol instead of the plant extract in the reaction system. A standard curve was established, using gallic acid as the standard [19].

### 2.9. Statistical Analysis

All measurements were conducted in three biological replicates, and results were expressed as mean ± standard deviation (n = 3). To evaluate statistically significant differences (*p* < 0.05), the Duncan method was applied together with One-Way ANOVA. A graphical representation of the data was achieved using Origin 2018 (OriginLab, Northampton, MA, USA), and statistical computations were conducted with SPSS 22.0 (IBM, Armonk, NY, USA).

## 3. Results

### 3.1. Effects of EBR Dosage on Watermelon Growth Under Cd Stress

**Figure 1:**
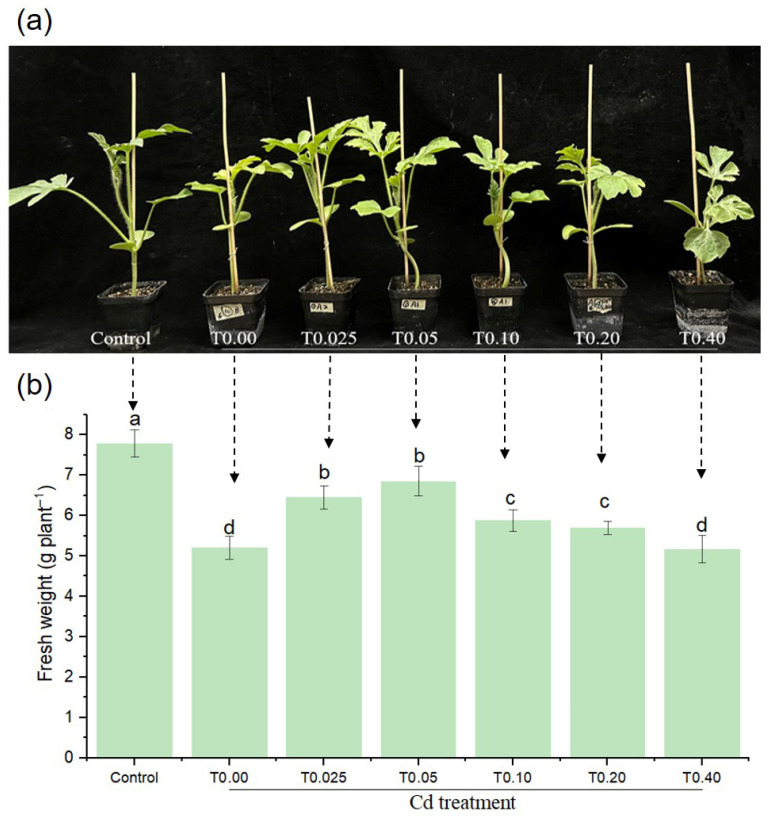
Effects of varying EBR concentrations on watermelon growth under Cd stress. Growth phenotype (**a**) and fresh weight (**b**). Note: Control plants were grown without Cd or EBR supplementation. Treatments included 0.2 mM Cd (as CdCl_2_·2.5H_2_O) and six EBR concentrations (0, 0.025, 0.05, 0.10, 0.20, and 0.40 μM), labeled T0.00 through to T0.40 on the *x*-axis. All data are presented as mean ± SD (n = 3 independent experiments), and statistically significant differences between groups are denoted by different letters (*p* < 0.05).

Watermelon seedlings exposed to Cd stress were treated with varying EBR concentrations (Figure 1). Compared to the Cd-free control, Cd treatment inhibited the growth of aboveground parts (Figure 1a), and the fresh weight (FW) decreased significantly by 33.06% (Figure 1b). The FW of seedlings pre-treated with different concentrations of EBR prior to Cd exposure was higher than that under Cd treatment alone, except for T0.40, with T0.05 (0.05 μM EBR) showing the greatest increase of 31.54%. These results indicated that EBR application effectively mitigated Cd toxicity in watermelon seedlings and promoted biomass accumulation, with the 0.05 μM offering the highest effect. Therefore, 0.05 μM EBR was selected for subsequent physiological and transcriptomic analyses.

### 3.2. Effects of EBR on Chlorophyll Levels and Cd Accumulation Under Cd Stress

**Figure 2:**
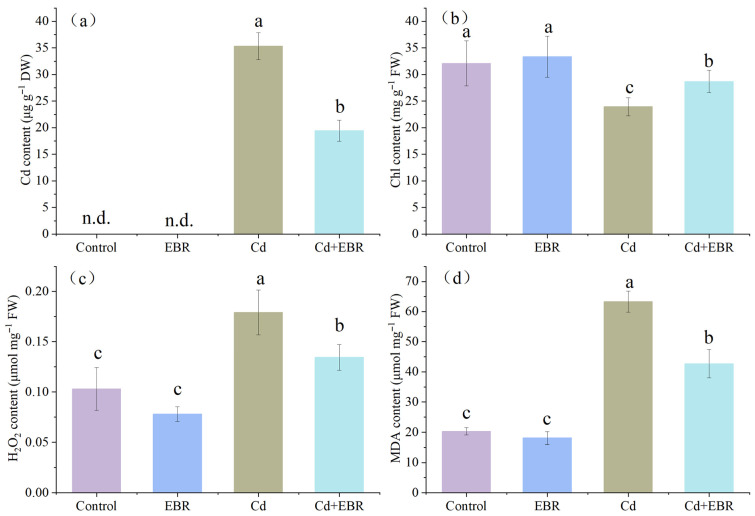
Effects of EBR treatment on the Cd accumulation (**a**), chlorophyll content (**b**), H_2_O_2_ production (**c**), and MDA (**d**) in the leaves of watermelon seedlings under Cd stress. Note: Control (no Cd, no EBR); EBR (0.05 µM EBR alone); Cd (0.2 mM Cd alone); and Cd + EBR (0.2 mM Cd + 0.05 µM EBR). All data are presented as mean ± SD (n = 3 independent experiments), and statistically significant differences between groups are denoted by different letters (*p* < 0.05).

Cd stress induced visible chlorosis in watermelon seedlings. To investigate this, the Cd accumulation in leaves was first quantified. As shown in Figure 2a, exposure to Cd caused a marked elevation in the leaf Cd concentration. However, foliar spray with 0.05 μM EBR significantly reduced Cd accumulation by 44.99% in comparison with plants given Cd alone. Chlorophyll is the pigment responsible for the green color of plants. Furthermore, Cd stress decreased the content of photosynthetic pigments by 25.44%, relative to the control (Figure 2b). Conversely, EBR treatment under Cd stress increased the pigment levels by 19.92% compared to non-EBR-treated, Cd-stressed plants. Notably, in the absence of Cd, EBR did not significantly affect the chlorophyll content.

### 3.3. Effects of EBR on H_2_O_2_ and MDA Accumulation Under Cd Stress

Cd exposure resulted in a significant increase in hydrogen peroxide (H_2_O_2_) levels and a rise in malondialdehyde (MDA) content, which serves as an indicator of membrane damage, compared with the control group (Figure 2c,d). As anticipated, foliar spraying of EBR effectively mitigated these adverse impacts: H_2_O_2_ levels in Cd-stressed plants were reduced by 32.54% following EBR treatment, relative to Cd-exposed plants without EBR (Figure 2c). Consistently, the MDA concentration in Cd-stressed watermelon seedlings decreased by 25.03% after EBR supplementation (Figure 2d), reflecting alleviated membrane lipid peroxidation. Notably, pretreatment with EBR alone had no significant effect on the H_2_O_2_ levels in un-stressed plants, but caused a clear reduction in the MDA content compared with the control group.

### 3.4. EBR-Mediated Regulation of Antioxidant Enzymatic Activities Under Cd Stress

**Figure 3:**
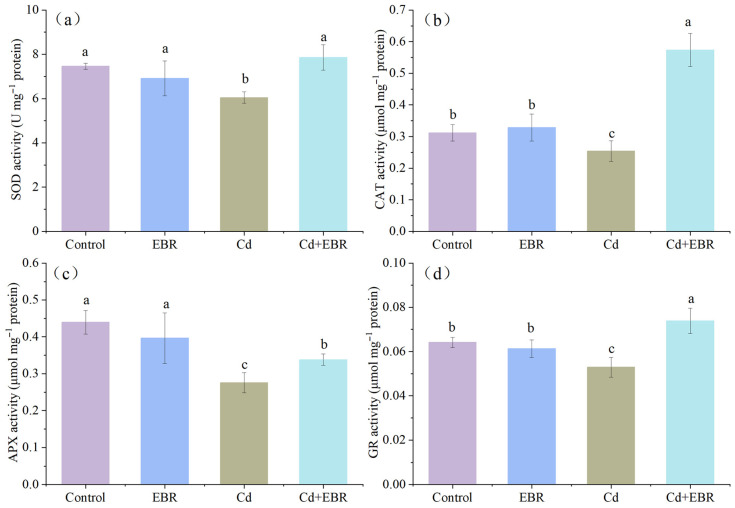
The effects of EBR treatment on the activities of superoxide dismutase (SOD) (**a**), catalase (CAT) (**b**), ascorbate peroxidase (APX) (**c**), and glutathione reductase (GR) (**d**) in the leaves of watermelon seedlings under Cd stress. Note: Control (no Cd, no EBR); EBR (0.05 µM EBR alone); Cd (0.2 mM Cd alone); and Cd + EBR (0.2 mM Cd + 0.05 µM EBR). All data are presented as mean ± SD (n = 3 independent experiments), and statistically significant differences between groups are denoted by different letters (*p* < 0.05).

Plants respond to oxidative stress by regulating the activities of antioxidant enzymes. As illustrated in Figure 3, Cd stress significantly inhibited the activities of key antioxidant enzymes in watermelon leaves, although the degree of inhibition varied. Compared with the control, Cd exposure markedly decreased the activities of superoxide dismutase (SOD), catalase (CAT), ascorbate peroxidase (APX), and glutathione reductase (GR) by 16.28% (Figure 3a), 18.58% (Figure 3b), 37.25% (Figure 3c), and 17.49% (Figure 3d), respectively. These results suggest that Cd imposes a strong inhibitory effect on the antioxidant defense system of watermelon seedlings. However, pretreatment with 2,4-epibrassinolide (EBR) markedly alleviated this inhibition. Compared with the Cd-only treatment, co-application of EBR and Cd significantly enhanced the activities of SOD, CAT, APX, and GR by 25.75%, 126.08%, 22.66%, and 39.57%, respectively. Under non-Cd stress conditions, EBR alone exerted no significant influence on the activities of these enzymes. Notably, under Cd stress, EBR supplementation not only restored but even elevated CAT and GR activities above the control levels, suggesting that EBR can effectively counteract Cd-induced inhibition and further enhance the activity of specific antioxidant enzymes in watermelon leaves.

### 3.5. RNA-Seq and Differential Gene Expression

To investigate the transcriptional regulatory mechanism by which EBR enhances Cd tolerance in watermelon, RNA sequencing (RNA-seq) was performed on leaf samples subjected to Cd treatment alone and in combination with EBR (Cd + EBR). Six libraries (three biological replicates per treatment group) were sequenced using the Illumina HiSeq platform. The raw data yielded an average base sum of 7,745,976,400 per sample. After quality filtering, an average of 25,819,921 clean reads per sample were retained. As shown in Table S2, the mean Q20, Q30, and GC contents were 95.6%, 87.6%, and 45.6%, respectively, indicating the high reliability and stability of the sequencing data. Additionally, the clean reads obtained from each sample were aligned to the watermelon reference genome with mapping efficiencies exceeding 92.7%, confirming the suitability of the reference genome for downstream analysis.

**Figure 4:**
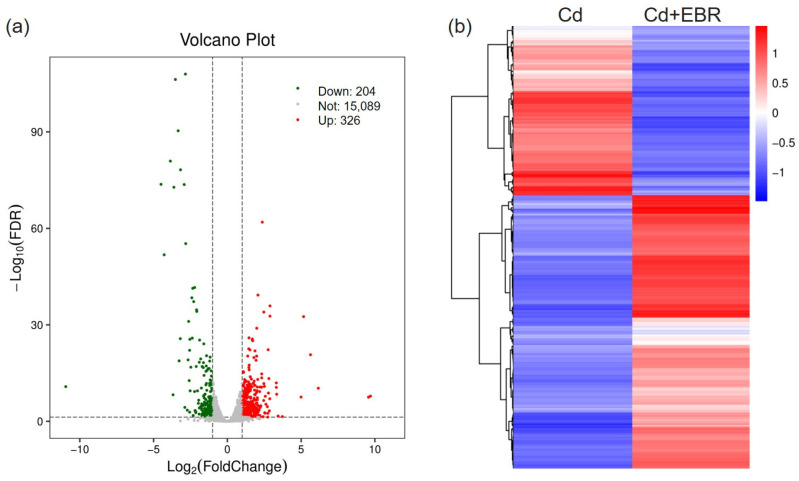
Transcriptome profiling and analysis of differentially expressed genes (DEGs) in watermelon leaves treated with Cd and Cd + EBR. (**a**) The volcano plot displayed the DEGs of two libraries. The significance of DEGs was defined with a false discovery rate (FDR) cutoff of <0.05. Genes that were up-regulated are represented by red dots, and those that were down-regulated by green dots, while transcripts that did not change significantly in the Cd + EBR library compared to Cd are represented by blue dots. (**b**) Based on log10 FPKM values, all DEGs were clustered hierarchically. The hue (blue to red) shows the intensity of gene expression from low to high. Cd and Cd + EBR represent two treatments under 0.2 mM Cd stress alone and 0.2 mM Cd stress with 0.05 µM EBR.

To identify genes involved in EBR-mediated Cd tolerance, differentially expressed genes (DEGs, Cd + EBR vs. Cd) were screened with the criteria of |log_2_(fold change)| ≥ 1 and q-value ≤ 0.05. A total of 530 DEGs were identified between the Cd and Cd + EBR groups (Table S3, Figure 4a). Hierarchical clustering analysis based on log10 FPKM values was performed for all DEGs, to visualize their expression patterns across the two groups (Figure 4b). Compared with the Cd treatment alone, the Cd + EBR treatment resulted in the upregulation of 326 DEGs and the downregulation of 204 DEGs. These results demonstrated that EBR significantly modulated the transcriptional levels of a specific group of genes in watermelon under Cd stress.

### 3.6. GO and COG Analysis of DEGs

To elucidate the functional roles of DEGs in watermelon in response to EBR treatment under Cd stress, Gene Ontology (GO) enrichment analysis was performed on the 530 DEGs identified from watermelon leaves (Figure S1a). These DEGs were categorized into 20 biological process (BP) terms, 11 molecular function (MF) terms, and 15 cellular component (CC) terms. Within the BP category, the most significantly enriched terms included “cellular process”, with “single-organism process”, “metabolic process”, and “response to stimulus” following closely. In the MF category, “catalytic activity” was the top enriched term, followed by “binding”, “transcription regulator activity”, and “transporter activity”. Within the CC category, the majority of DEGs were assigned to terms such as “cell part”, “cell”, “organelle”, and “membrane”.

To further explore the functional classifications of these DEGs, clusters of orthologous groups (COG) analysis was carried out to annotate the functional categories of the DEGs (Figure S1b). The DEGs were grouped into 20 functional categories, with the most represented being “posttranslational modification, protein turnover, chaperones”, “amino acid transport and metabolism”, “general function prediction only”, and “carbohydrate transport and metabolism”.

To pinpoint the key functional categories modulated by EBR, separate enrichment analyses were performed for total, up- and down-regulated DEGs (Table S3). For total DEGs, the GO enrichment analysis reveals that 24-EBR treatment under Cd stress primarily activates biological processes related to phenylpropanoid metabolism, including cinnamic acid biosynthesis, L-phenylalanine catabolism and biosynthesis, and aromatic amino acid biosynthesis, along with galactose metabolism, cell wall biogenesis, and response to water deprivation. The cellular components enriched include the extracellular region and space, glyoxysome, and plant-type cell wall, while the molecular functions highlight phenylalanine ammonia-lyase (PAL) activity, inositol 3-alpha-galactosyltransferase activity, arogenate dehydratase activity, and heme and iron ion binding. These results further confirm that EBR enhances cadmium tolerance by activating the phenylpropanoid pathway, modifying cell wall properties, and regulating stress-responsive metabolic processes.

### 3.7. KEGG Pathway Analysis of the DEGs

**Figure 5:**
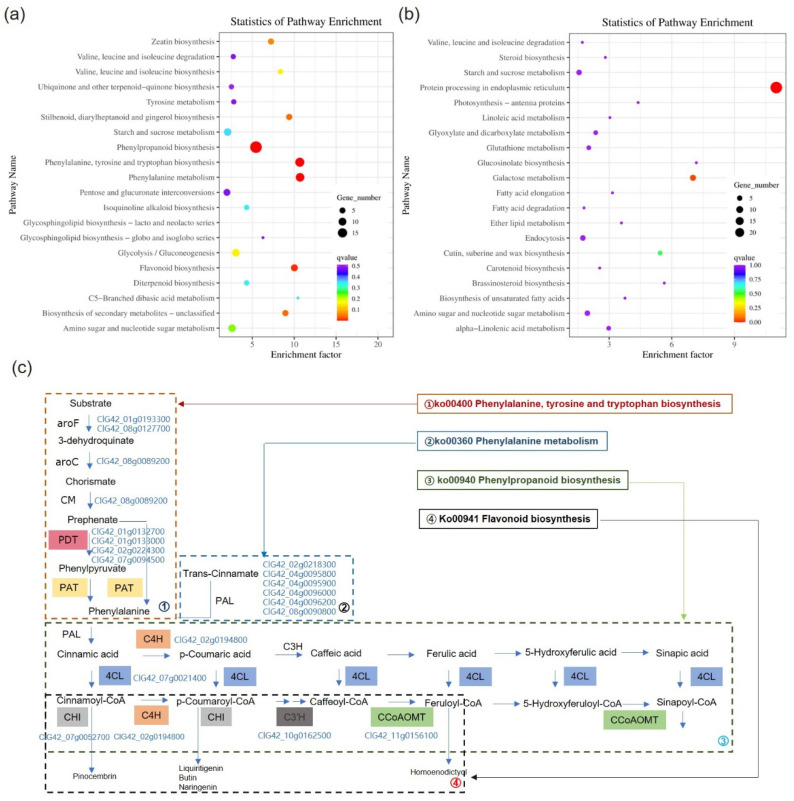
Analysis of KEGG enrichment between treatment with Cd and treatment with Cd + EBR on watermelon leaves. The top 20 significantly enriched KEGG pathways for up-regulated (**a**) and down-regulated (**b**) differentially expressed genes (DEGs) are presented. KEGG pathways are shown on the left Y-axis, and the Rich factor is plotted on the X-axis. The color gradient and dot size represent the range of q-values and the number of DEGs assigned to each pathway, respectively, which is displayed as a color scale. DEGs that are active in the phenylpropanoid pathway (**c**). DEGs controlled by EBR are involved in the synthesis of phenylalanine, tyrosine, tryptophan (ko00400), phenylpropanoid (ko00940), phenylalanine (ko00360), and flavonoid (ko00941). The four pathways (Ko00941, ko00400, Ko00940, and Ko00360) each entail a metabolic pathway that is mediated by PDT (arogenate/prephenate dehydratase), PAT (Phenylalanine/tyrosine aminotransferase or Prephenate aminotransferase), PAL (phenylalanine ammonium lyase), C4H (cinnamic acid 4-hydroxylase), 4CL (4-coumarate-CoA ligase), CHI (chalcone isomerase), C3H (p-coumarate 3-hydroxylase), and CCoAOMT (caffeoyl-CoA O-methyltransferase). The up-regulated DEGs are presented in the blue font. Map00400 provides phenylalanine skeleton via PDT/PAT; sequential action of PAL, C4H, 4CL (map00940) yields intermediates that either enter the flavonoid pathway via CHS+CHI (map00941 → anthocyanins/flavonols) or undergo C3H/CCoAOMT-mediated modification toward monolignols, while map00360 competitively degrades phenylalanine.

Pathway analysis of up- and down-regulated DEGs was carried out by utilizing the KEGG pathway database with KOBAS to further investigate the metabolic processes that are regulated by EBR (Figure 5). The six KEGG pathways: phenylpropanoid biosynthesis (map00940), Phenylalanine, tyrosine and tryptophan biosynthesis (map00400), phenylalanine metabolism (map00360), flavonoid biosynthesis (map00941), stilbenoid, diarylheptanoid, gingerol biosynthesis (map00945), and biosynthesis of secondary metabolites—unclassified (map00999) were all significantly enriched in up-regulated DEGs (Figure 5a). The galactose metabolism (map00052) and protein processing in the endoplasmic reticulum (map04141) were both considerably enriched in the down-regulated DEGs (Figure 5b). Interestingly, five pathways—map00940, map00400, map00360, map00941 and map00945—were connected to the phenylpropanoid pathway and the derived metabolic network (Figure 5c). Map00400 provides the only carbon skeleton for phenylpropanoid metabolism—phenylalanine, which is deaminated by phenylalanine ammonia lyase (PAL) to produce cinnamic acid, which is then activated by 4-coumaroyl CoA synthase (4CL) as a key intermediate to drive phenylpropanoid biosynthesis (map00940). The intermediate further condenses with acetyl CoA under the catalysis of chalcone synthase (CHS), followed by CHI to generate naringenin, initiating the biosynthesis of flavonoids (map00941), forming an irreversible cascade from aromatic amino acids to secondary metabolites such as anthocyanins and flavonols. Phenylalanine metabolism (map00360), as a competitive collateral branch, mainly mediates the degradation of phenylalanine.

### 3.8. qRT-PCR Validation

Quantitative real-time PCR (qRT-PCR) analysis was performed on 12 randomly selected DEGs to validate the accuracy of the transcriptome data showing diverse expression patterns from two watermelon seedling treatments, with actin serving as the housekeeping gene (Figure S2). Most genes exhibited transcriptional profiles that were consistent with the RNA-seq data, and the strong correlation coefficient (R^2^ = 0.9683) illustrated the high robustness of the transcriptome results.

### 3.9. EBR Regulates Phenylpropanoid Biosynthesis

**Figure 6:**
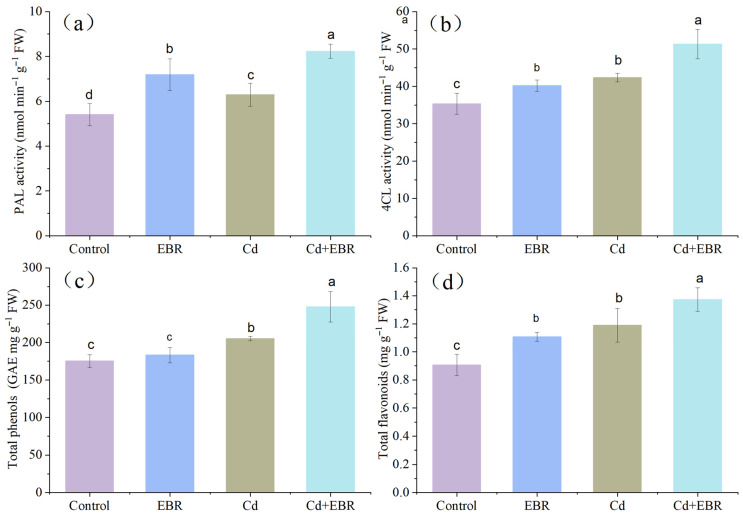
Effects of EBR treatment on the activities of PAL (**a**) and 4CL (**b**), as well as contents of phenol (**c**) and flavonoid (**d**) in the leaves of watermelon seedlings exposed to Cd stress. Note: Control (no Cd, no EBR); EBR (0.05 µM EBR alone); Cd (0.2 mM Cd alone); and Cd + EBR (0.2 mM Cd + 0.05 µM EBR). All data are presented as mean ± SD (n = 3 independent experiments), and statistically significant differences between groups are denoted by different letters (*p* < 0.05).

The most significantly enriched pathway among the DEGs was “Phenylpropanoid biosynthesis” (ko00940), which contained 15 up-regulated genes, as shown in Figure 5 and Table S4. The following enzymes are involved in this pathway (Figure 5c): 5-O-(4-coumaroyl)-D-quinate 3′-monooxygenase (CYP98A, C3′H, EC 1.14.14.96), 4-coumarate-CoA ligase (4CL, EC 6.2.1.12), phenylalanine ammonia-lyase (PAL, EC 4.3.1.24), cinnamic acid 4-hydroxylase (C4H, EC 1.14.13.11), and caffeoyl-CoA O-methyltransferase (cCoAOMT, EC 2.1.1.104). Under Cd stress, EBR increased the expression of the *PAL*, *4CL*, *C4H*, *C3′H*, and *cCoAOMT* genes (Figure S2). To further support the involvement of this pathway, the activities of two crucial enzymes, PAL and 4CL, were examined in this case. Compared with Cd stress alone, EBR increased PAL and 4CL activities in watermelon leaves under Cd stress by 30.82% (Figure 6a) and 21.03% (Figure 6b), respectively.

Phenylpropanoid biosynthesis is derived from phenylalanine, and several secondary metabolites, including flavonoids and phenolic compounds, are synthesized in this pathway. Accordingly, we further measured the contents of these metabolites. Compared with Cd treatment alone, EBR promoted the accumulation of these compounds in watermelon leaves, with the total phenolic and total flavonoid contents increasing by 20.68% (Figure 6c) and 15.41% (Figure 6d), respectively. These results demonstrate that EBR activates the phenylpropanoid biosynthesis pathway and enhances Cd tolerance in watermelon through the regulation of key gene expression, enzyme activity, and the accumulation of related secondary metabolites.

## 4. Discussion

### 4.1. EBR Mitigates Cd-Caused Growth Inhibition of Watermelon

Cd is not an essential nutrient for plant growth. When accumulated to a certain concentration in plant tissues, Cd induces a series of toxic symptoms including leaf chlorosis, growth retardation, biomass, yield and quality decline [3,4,5,20,21]. EBR is a sterol plant hormone, which can stimulate the intrinsic potential of plants. It plays a vital role in regulating plant growth and development by impacting plant cell division and growth, raising plant metabolism, and enhancing the stress tolerance of crops [6,9,11]. Our results confirmed that Cd stress severely impaired watermelon growth and induced chlorophyll degradation (Figure 1 and Figure 2), consistent with previous reports [15]. Spraying exogenous EBR could effectively alleviate the influence of Cd on watermelon seedlings, increase their FW, reduce the accumulation of Cd, and increase the content of chlorophyll, thus promoting the growth of watermelon seedlings, which agrees with the previous findings [2,16]. EBR can promote plant growth, probably because it can regulate cell wall synthesis and modification, stimulate ATPase activity, pump a large amount of H^+^ into the cell wall, increase its plasticity, and affect cell elongation and division [22]. Cd content in Kentucky bluegrass roots considerably decreased through EBR application [10]. In the present study, exogenous EBR inhibited the accumulation of Cd in watermelon leaves (Figure 2). As reported in many studies, BR application enhances the absorption of various cations, such as K^+^, Ca^2+^, and Mg^2+^, in plant roots. These cations are subsequently preferentially transferred by stellar bundles to the immature tissues found in the leaves, lowering Cd uptake [8]. The reason might be that BRs play a role in the transport of Cd from roots to stems in plants through regulating the ion absorption and maintaining ion homeostasis through the root to lessen the uptake of heavy metals. Here, the mechanism of EBR on Cd accumulation and transport in watermelon seedlings needs to be further explored. Exogenous application of EBR enhances the chlorophyll content and photosynthetic efficiency in plants under metal stress, which likely contributes to improved growth [23]. In this experiment, spraying an appropriate amount of EBR on the leaves could effectively improve the biomass and photosynthetic pigment content of watermelon with Cd stress, indicating that EBR may enhance the ability of watermelon seedlings to accept Cd stress. This may be attributed to EBR stimulating the activity of certain enzymes involved in chlorophyll biosynthesis, or inducing the expression of specific genes encoding chlorophyll synthases [9]. Recently, a transcriptome study found that under Cd stress, EBR treatment could significantly upregulate the expression levels of chlorophyll biosynthesis-related genes, conversion, and degradation, maintaining the stability of the photosynthetic system by enhancing the chlorophyll cycle [24].

### 4.2. EBR Alleviates Cd-Caused Oxidative Damage in Watermelon

When heavy metal stress causes plants to suffer, a large amount of reactive oxygen species (ROS) are produced, leading to lipid peroxidation. MDA serves as the end product of lipid peroxidation of the cell membrane, and its level always indicates the degree of lipid peroxidation. Antioxidant enzymes including SOD, CAT, APX, and GR can get rid of ROS to protect cell membranes from ROS damage [10,11,20,21]. Vitis vinifera seedlings under Cd stress exhibited a significant increase in ROS, MDA content, and membrane permeability [25]. In contrast, the application of EBR at a specific concentration significantly boosted antioxidant enzyme activities and dramatically decreased both the MDA content and plasma membrane permeability. In this investigation, the contents of H_2_O_2_ and MDA in watermelon seedling leaves were significantly increased under Cd stress. However, following the foliar application of EBR, both parameters decreased significantly (Figure 2). This mitigating effect is also consistent with previous findings in other species, such as *Brassica juncea* [16] and *Vigna angularis* [11], demonstrating that exogenous EBR alleviates Cd-induced oxidative damage. Specifically, EBR application reduces membrane permeability and ROS accumulation, thereby stabilizing membrane structure and promoting seedling growth under Cd stress.

The observed reduction in oxidative damage is likely attributable to the activation of antioxidant enzymes by EBR. Numerous studies have reported that exogenous EBR significantly enhance the activities of key antioxidant enzymes (SOD, CAT, APX, and GR), thereby effectively mitigating Cd-induced oxidative damage in watermelon seedlings [2,11,16,25]. For instance, in *Brassica juncea*, foliar EBR application boosts the activities of SOD, CAT, APX, and GR, reducing Cd-induced damage [16]. Similarly, it was reported that Cd stress led to an increase in MDA and H_2_O_2_ levels in Kentucky bluegrass roots, while the application of EBR consistently enhanced SOD, CAT, and GR activities, resulting in decreased ROS and MDA accumulation [10]. In agreement with these results, our study found that Cd treatment significantly decreased the activities of key antioxidant enzymes (SOD, GR, APX, and CAT) in watermelon leaves, whereas subsequent EBR application enhanced these enzyme activities to varying degrees (Figure 3). This suggests that EBR, as a bioactive compound, activates the plant antioxidant defense system to scavenge excessive ROS and thereby alleviates Cd-induced oxidative damage and enhances Cd resistance in watermelon seedlings.

### 4.3. EBR Activates Cell Wall Biogenesis and Inhibits Protein Processing in the ER in Watermelon

Recently, RNA-seq analysis has shown that EBR can regulate the uptake, transport, and accumulation of Cd^2+^ in adzuki bean seedling roots, thereby alleviating the toxic damage of Cd to root cells [11]. In the present study, to elucidate EBR-mediated transcriptional regulation in watermelon under Cd stress, the transcriptome analysis was performed, identifying 530 DEGs regulated by EBR (Figure 4). However, GO and KEGG enrichment analyses demonstrated that these DEGs were mainly associated with phenylpropanoid biosynthesis, endoplasmic reticulum’s protein production, cell wall organization, etc. DEGs in the phenylpropanoid pathway may enhance plant tolerance to Cd stress by modulating the lignification of cell walls and the biosynthesis of antioxidant secondary metabolites [21]. This also verifies that EBR regulates antioxidant regulation under Cd stress, and many DEGs are clustered in the extracellular/cellular component, which is related to cell wall biogenesis and organization (Table S3). To guarantee optimal cell wall properties across various environments, the BR signaling pathway adjusts the balance between cell wall stiffening and loosening [26]. The main components of plant cell walls are important for cadmium (Cd) fixation. Six cell wall synthesis-related genes were identified (Table S3) and classified into Glycosyltransferases (ClG42_01g0267600), Cellulose synthase (ClG42_10g0111300), and Xyloglucan endotransglycosylases/XTH (ClG42_10g0083600, ClG42_10g0130500, ClG42_09g0225300, ClG42_10g0083900). Previous studies have underscored the pivotal role of XTH in the responses of plants to heavy metal stress [27]. XTH, a pivotal enzyme that cleaves and reattaches xyloglucan chains to remodel plant cell walls, is up-regulated by EBR to reinforce Cd immobilization via enhanced hemicellulose network integrity, thereby establishing a robust first-line physical barrier against metal toxicity. BR may control the transcript level of several *XTHs* or *EXPANSIN* in rice, wheat, and maize [26]. Recently, it was found that 12 DEGs induced by EBR under zinc stress were clustered into the protein processing in the endoplasmic reticulum (ER) in watermelon [28]. However, in this study, it was found that all DEGs involved in this pathway were down-regulated by EBR under Cd stress (Table S4). It was reported that Cd stress triggers ER stress and the subsequent unfolded protein response (UPR) in Arabidopsis. Also, Cd stress reduces the core receptor BRI1 protein levels in the BR signaling pathway [29]. A UPR-deficient mutant (*bzip28/bzip60*) maintains normal BRI1 levels and exhibits stronger Cd tolerance, suggesting that BRI1 stability is key for adaptation. The presence of BRI1 glycoprotein in the *bzip28/bzip60* mutant can at least partially account for the enhanced adaptability of this mutant under Cd stress [29]. Therefore, it is speculated that brassinosteroids do not simply superimpose “stress signals”, but maintain the normal transmission of BR signals by protecting the core components like BRI1 receptors [30]. Typically, BiP, bZIP60, HSP70, and HSP20 are core marker genes involved in regulating endoplasmic reticulum homeostasis and stress response across various biological systems. Here, only HSP70 and HSP20 were found to cluster under ER stress. In contrast to the conventional view that HSP70 upregulation enhances stress tolerance [28], the present transcriptomic data revealed a significant downregulation of several HSP70-encoding genes within the endoplasmic reticulum protein processing pathway upon EBR treatment under Cd stress. In *Arabidopsis thaliana*, AtHsc70-1 negatively regulates the basal heat tolerance through affecting the activity of HsfAs and Hsp101 [31]. AtHsp70-15-deficient plants are more tolerant to infection by turnip mosaic virus [32]. Hsp70 negatively regulates autophagy in NSCLC cells [33] and EBR was reported to promote autophagic activity, which contributed to the degradation of damaged chloroplasts under drought stress [34]. Whether EBR enhances Cd tolerance via modulating autophagy in watermelon or other plants remains elusive and warrants further investigation.

### 4.4. EBR Activates Phenylpropane Metabolism and Phenylpropanoid Biosynthesis in Watermelon

Phenylpropane metabolism pathway is the main source of numerous defensive secondary metabolites in plants, including polyphenols, flavonoids, and lignin, and it forms a complex network regulated by a variety of enzymes, including cinnamate-4-hydroxylase (C4H), 4CL, PAL, caffeic acid 3-O-methyltransferase (CCoAOMT), etc. [35]. The phenylpropanoid biosynthesis pathway has been implicated in the responses of plants to heavy metal stress. For instance, in Cd-exposed wheat roots, the phenylpropanoid biosynthesis pathway was uniquely enriched. Genes encoding enzymes including PAL, 4CL, CCR, CALDH, and CAD are involved in the biosynthesis of flavones, isoflavones, and lignin, which are compounds that are potentially associated with Cd tolerance [36]. Similarly, under Zn stress, phenylpropanoid biosynthesis was significantly enriched in *Citrullus lanatus* L., and the gene expression levels of *PAL*, *4CL*, *CCR*, and *CCoAOMT* were significantly induced after EBR treatment [28]. In this study, KEGG enrichment analysis of DEGs from EBR-treated watermelon leaves under Cd stress revealed significant enrichment in the “phenylpropanoid biosynthesis” pathway (map00940), as well as its upstream precursor pathway, “phenylalanine, tyrosine and tryptophan biosynthesis” (map00400), and the downstream “flavonoid biosynthesis” pathway (map00941). These findings suggest that EBR may alleviate Cd toxicity in watermelon through the modulation of phenylpropanoid metabolism. Phenylalanine is the precursor to the production of phenylpropanoid compounds.

p-Coumaroyl-CoA is converted into caffeoyl-CoA and feruloyl-CoA through a series of enzymatic reactions catalyzed by key enzymes in the phenylpropanoid pathway, including4CL, C4H, HCT, and CCoAOMT [35]. As the entry-point enzyme, PAL is critical for channeling the carbon flow into the phenylpropanoid pathway, providing substrates for lignin and flavonoid biosynthesis. Notably, PAL expression has been directly correlated with heavy metal accumulation [37]. In line with this, we identified six up-regulated PAL transcripts in EBR-treated watermelon leaves under Cd stress (Figure 5c, Table S4), which may contribute to enhanced PAL activity and thereby promote the synthesis and accumulation of downstream secondary metabolites [38]. Additionally, single up-regulated sequences encoding 4CL, C4H, and CCoAOMT were identified, potentially enhancing the metabolic flux through the entire pathway. The increased activities of PAL and 4CL further corroborated the regulatory role of this pathway (Figure 6).

Phenylpropanoid biosynthesis produces a diverse array of phenolic compounds, including flavonoids, coumarins, hydrolysable tannins, monolignols, lignans, and lignins, which protect plants against abiotic stress through their antioxidative capacity, participation in metal chelation, and ROS scavenging [39]. The key genes involved in phenylpropanoid biosynthesis are regulated by *Bacillus altitudinis* WR10, which enhances the accumulation of phenolic acids and protects plant cells against copper toxicity [40]. Flavonoids, a specific type of phenolic compound and an important class of stress-resistant substances, play a protective antioxidant role in plant tolerance to heavy metal stress [41]. The biosynthesis of flavonoids involves several enzymes, including CHS, F3H, and chalcone isomerase (CHI). In this study, a total of four differentially up-regulated genes encoding four key enzymes were identified in watermelon leaves (Figure S2; Table S4). Among these, one enzyme (C4H) is required for the core phenylpropanoid pathway, and three enzymes (CHI, C3′H, and CCoAOMT) are involved in flavonoid biosynthesis. Additionally, under Cd stress, EBR promoted the accumulation of the total phenolics and total flavonoids in watermelon leaves (Figure 6). The upregulation of the corresponding genes and enzymes may mediate the Cd accumulation and detoxification in leaves. Exogenous EBR also effectively activates secondary metabolism in salt-stressed rice by promoting the expression of genes related to carotenoid and flavonoid biosynthesis [41]. Moreover, phenolic compounds exert a dual function in alleviating heavy metal stress through direct ROS scavenging and metal chelation [42]. Overall, our findings indicate that the phenylpropanoid biosynthesis pathway and its derived metabolic networks are key components of EBR-induced Cd tolerance in watermelon, given their central roles in the metabolic network regulated by EBR. The upregulation of genes involved in phenylalanine metabolism, phenylpropanoid biosynthesis, and flavonoid biosynthesis collectively promotes the accumulation of secondary metabolites such as phenols and flavonoids. On one hand, these compounds act as potent antioxidants that directly scavenge excessive reactive oxygen species induced by Cd stress, thereby alleviating membrane lipid peroxidation and cellular damage. On the other hand, intermediate products of phenylpropanoid metabolism participate in cell wall lignification, enhancing the adsorption and immobilization of Cd ions in the cell wall and reducing Cd transport to the shoots. Furthermore, flavonoids can chelate Cd, further reducing its toxicity. Therefore, the coordinated activation of these pathways represents an important molecular defense mechanism in watermelon seedlings for alleviating Cd toxicity. Future investigations should focus on elucidating the specific molecular mechanisms by which EBR modulates these pathways to enhance plants’ defense against Cd toxicity.

## 5. Conclusions

**Figure 7:**
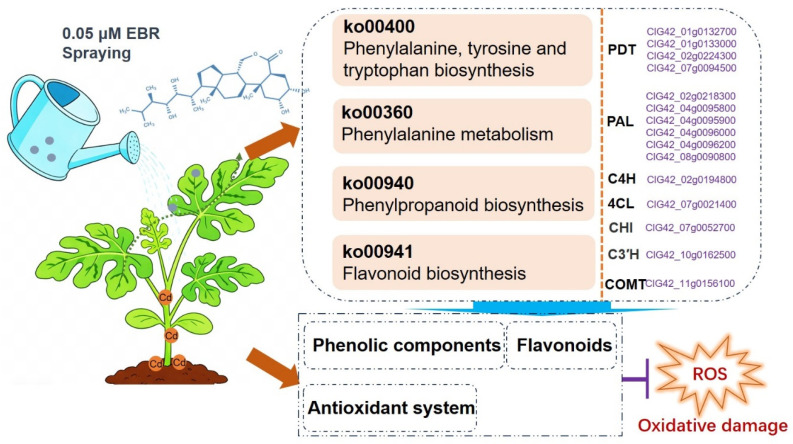
Schematic diagram of the potential mechanism of EBR alleviating Cd stress in watermelon. EBR regulates the secondary metabolism pathways in watermelon seedlings under Cd stress, specifically the phenylpropanoids and their derived metabolic networks, starting from aromatic amino acids. Arogenate/prephenate dehydratase (PDT), Phenylalanine ammonium lyase (PAL), cinnamic acid 4-hydroxylase (C4H), 4-coumarate-CoA ligase (4CL), 5-O-(4-coumaroyl)-D-quinate 3′-monooxygenase (C3′H) Caffeic acid-O-methyltransferase (COMT), 24-epibrassinolide (EBR) and cadmium (Cd).

This research demonstrates that the exogenous application of EBR effectively enhances Cd tolerance in watermelon seedlings. The protective effect is achieved by reducing Cd accumulation, preserving chlorophyll, and alleviating oxidative stress. EBR not only boosts the activity of key antioxidant enzymes but also activates the phenylpropanoid pathway to synthesize a suite of secondary metabolites like phenolic and flavonoid components that provide sustained antioxidant protection (Figure 7). These findings elucidate the physio-biochemistry and transcriptome regulatory network underlying EBR-mediated Cd tolerance and provide a basis for breeding heavy-metal-tolerant watermelon cultivars.

## Data Availability

The entire data presented in this study are available upon request from the corresponding author.

## Supplementary Materials

The following supporting information can be downloaded at: https://www.mdpi.com/article/10.3390/biology15080638/s1, Table S1: Primers for qRT-PCR; Table S2: Summary of RNA-seq read assembly, Table S3: Gene ontology (GO) enrichment analysis of differentially expressed genes (DEGs) in Cd and Cd + EBR treatment of watermelon leaves; Table S4: Kyoto encyclopedia of genes and genomes (KEGG) enrichment analysis of differentially expressed genes (DEGs) in Cd and Cd + EBR treatment of watermelon leaves. Figure S1: Gene ontology (GO) and clusters of orthologous groups (COG) classification of differentially expressed genes (DEGs) in watermelon leaves treated with Cd and Cd + EBR. (a) 530 DEGs were selected for GO annotation, covering three terms: molecular function, biological process, and cellular component. (b) All DEGs were selected for functional classification by cluster of orthologous groups. Figure S2: QRT-PCR and Pearson’s correlation of RNA-seq. (a) Verification using qRT-PCR of differentially expressed genes (DEGs). qRT-PCR was used to examine 12 DEGs, including *PAT* (ClG42_07g0094500); *CM* (ClG42_11g0060600); *PAL* (ClG42_02g0218300); *CYP73A* (ClG42_02g0194800); *4CL* (ClG42_07g0021400); *cCoAOMT* (ClG42_11g0156100); UGT72E (ClG42_07g0092700); *CHI* (ClG42_07g0052700); *HSP70* (ClG42_11g0131800); *IFF6* (ClG42_03g0172100); *HSP20* (ClG42_11g0131800); *IQM2* (ClG42_03g0127300. (b) Results of RNA-seq and QRT-PCR were correlated using Pearson’s formula.
