# Supplementary material for: Integrated Physio-Biochemistry and Transcriptome Analysis Reveals the Mechanism of 24-Epibrassinolide in Alleviating Cadmium Stress in Watermelon (Citrullus lanatus L.)
Source: Biology (Basel). 2026 Apr 18;15(8):638. doi: 10.3390/biology15080638 (PMC13113492; doi:10.3390/biology15080638)
Supplement: Supplementary file 1 [file biology-15-00638-s001.zip › biology-4231271-supplementary-Figure.pdf]

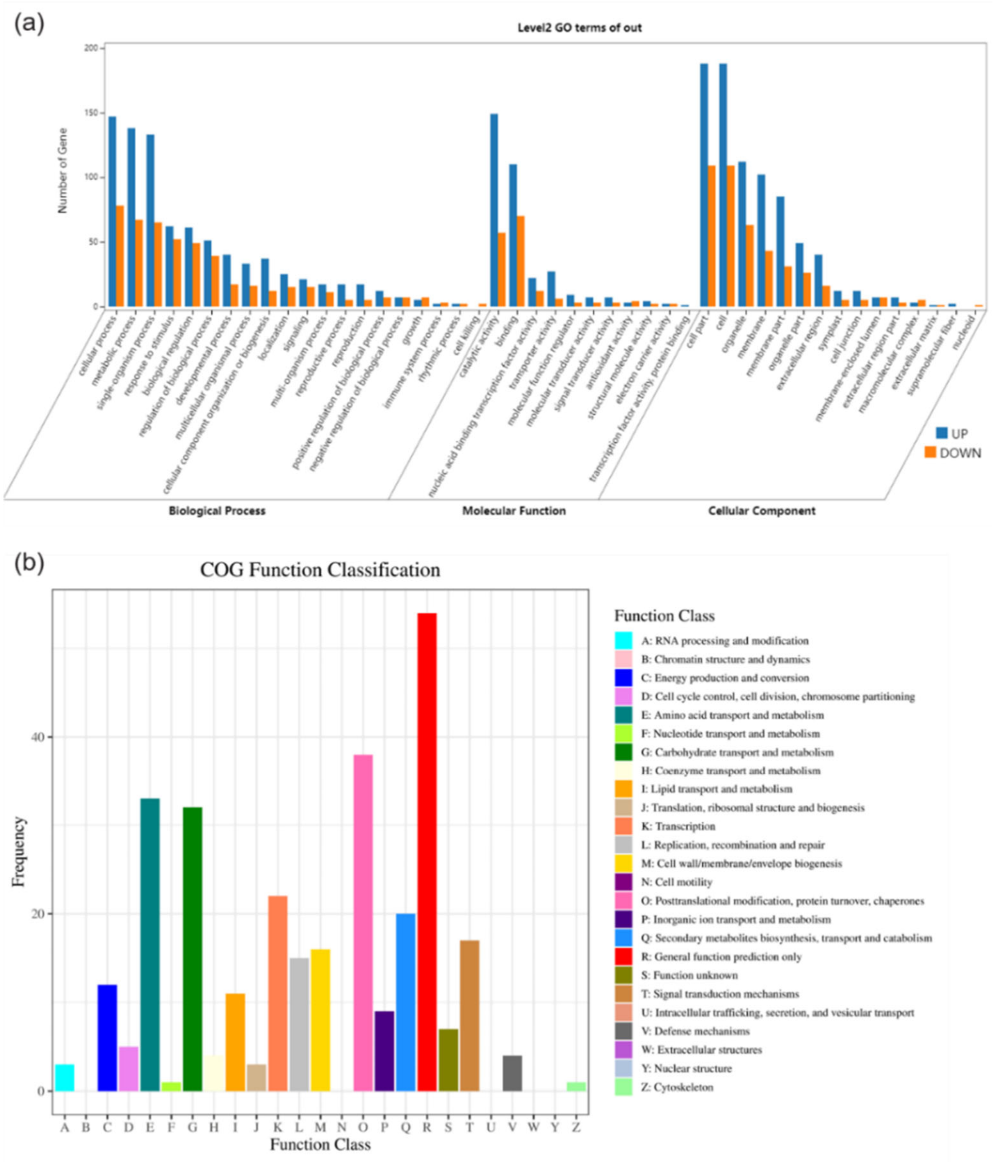

Figure S1. Gene ontology (GO) and clusters of orthologous groups (COG) classification of differentially expressed genes (DEGs) in watermelon leaves treated with Cd and Cd+EBR. (a) 530 DEGs were selected for GO annotation, covering three terms: molecular function, biological process, and cellular component. (b) All DEGs were selected for functional classification by cluster of orthologous groups.

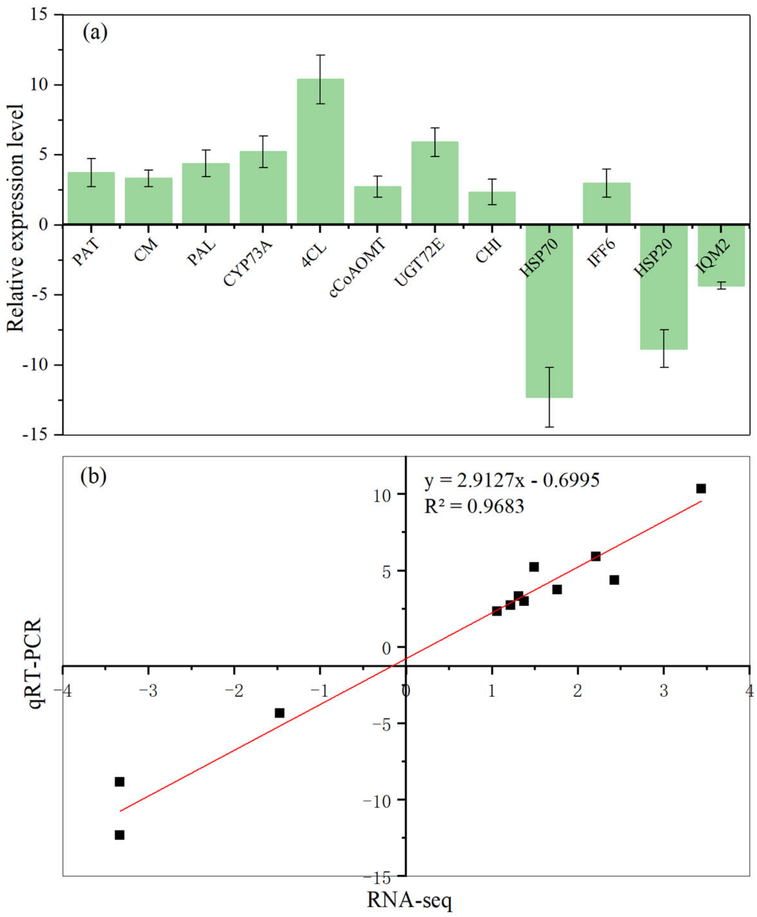

Figure S2. QRT-PCR and Pearson’s correlation of RNA-seq. (a) Verification using qRT-PCR of differentially expressed genes (DEGs). qRT-PCR was used to examine 12 DEGs, including *PAT* (ClG42_07g0094500); *CM* (ClG42_11g0060600); *PAL* (ClG42_02g0218300); *CYP73A* (ClG42_02g0194800); *4CL* (ClG42_07g0021400); *cCoAOMT* (ClG42_11g0156100); UGT72E (ClG42_07g0092700); *CHI* (ClG42_07g0052700); *HSP70* (ClG42_11g0131800); *IFF6* (ClG42_03g0172100); *HSP20* (ClG42_11g0131800); *IQM2* (ClG42_03g0127300. (b) Results of RNA-seq and QRT-PCR were correlated using Pearson's formula.
